# Effects of Tilmicosin Treatment on the Nasopharyngeal Microbiota of Feedlot Cattle With Respiratory Disease During the First Week of Clinical Recovery

**DOI:** 10.3389/fvets.2020.00115

**Published:** 2020-02-28

**Authors:** Mohamed Zeineldin, James Lowe, Brian Aldridge

**Affiliations:** ^1^Carl R. Woese Institute for Genomic Biology, University of Illinois at Urbana-Champaign, Urbana, IL, United States; ^2^Department of Animal Medicine, College of Veterinary Medicine, Benha University, Benha, Egypt; ^3^Integrated Food Animal Management Systems, Department of Veterinary Clinical Medicine, College of Veterinary Medicine, University of Illinois at Urbana-Champaign, Urbana, IL, United States

**Keywords:** respiratory disease, feedlot, microbiota, tilmicosin, 16S rRNA gene

## Abstract

While the nasopharyngeal (NP) microbiota is believed to be a key player in bovine respiratory health, there is limited published information about the change of NP microbiota associated with clinical recovery from bovine respiratory disease (BRD). The objective of this study was to evaluate the effect of tilmicosin treatment on the NP microbiota composition and diversity of BRD-affected calves during the first week of clinical recovery. Deep NP swabs were collected from diseased calves at the initial diagnosis of BRD, and again 7 days after the administration of a single dose of tilmicosin. As an experimental control, samples were collected from clinically healthy, pen-matched calves at the time of initial BRD diagnosis. In general, the NP microbiota from the control calves were more diverse than the NP microbiota from tilmicosin treated and BRD-affected calves. Principle coordinate analysis (PCOA) of Bray-Curtis and Jaccard dissimilarity also revealed that the overall composition of NP microbial communities in tilmicosin-treated calves closely resembled that of BRD-affected calves but differed significantly from pen-matched healthy calves. Overall, it appeared that there were only minor changes in NP microbial communities following tilmicosin treatment and, during the early phase of clinical recovery the NP microbiota in treated animals was disparate from that observed in healthy control calves. Understanding the potential impact of this prolonged recovery in mucosal microbiota would be important in optimizing the use of antimicrobials in health management programs in the feedlot industry.

## Introduction

Bovine respiratory disease is a common and costly health failure associated with a polymicrobial infection often occurring in newly transported feedlot cattle (1, 2). Various predisposing factors, such as neurohumoral stress, nutritional changes, environmental conditions, and upper respiratory mucosal damage from viral and bacterial pathogens have been implicated in the pathogenesis of BRD (3, 4). Clinical research shows that the most common bacterial pathogens associated with BRD are known to be normally transient residents of the upper respiratory tract of healthy cattle that become opportunistic pathogens when viral infection and various management stressors combine to impair the host immune system (5, 6). The careful use of antimicrobials for prophylaxis, metaphylaxis and therapy has offered significant advances in BRD management in high-risk cattle (3). Tilmicosin is a long-acting macrolide with strong bactericidal action that can protect the cattle against BRD pathogens for up to 7 days (7) and, given its favorable bioavailability and broad efficacy against many BRD pathogens, is a popular option for the treatment and prevention of BRD (8). Feedlot cattle at high-risk of developing respiratory disease, and treated with tilmicosin, showed a distinct shift in the composition of NP microbiota during the first 10 days after arrival on the farm (7). Tilmicosin also markedly reduced the prevalence of microbes in the nasal secretions of BRD-affected calves for up to 6 days compared to control calves (9). With increasing concerns regarding the overall efficacy of current antibiotic treatment approaches and the growing emergence of antimicrobial resistance (10), new management strategies for optimizing mucosal health and immune defenses are required. Moreover, understanding the impact of infectious disease processes and antimicrobial agents on the respiratory microbial ecosystem is important clinically, since these communities appear to have a crucial role in maintaining mucosal health (11). The objective of this study was to evaluate the effects of tilmicosin treatment on the composition and diversity of the NP microbiota of BRD-affected calves during the first week of clinical recovery.

## Materials and Methods

### Study Populations and Sample Collection

This study was a part of a larger experiment that examined the clinical and microbial predictors of susceptibility to BRD in beef cattle (12, 13). The use of the animals, and all experiments procedures were performed in accordance with relevant guidelines, and under the approval of, the University of Illinois Institutional Animal Care and Use Committee (IACUC Protocol: #15064). Briefly, a total of 135, 6 to 8-month-old, single source, Charolais feedlot calves (mean entry body weight 247 ± 33.8 kg) from the commercial and research university feedlot at South Farms Beef cattle and Sheep Field Laboratory (Urbana, IL, USA) were involved in this study. All calves were processed within 24 h after arrival to the farm. During the first month after arrival, all calves were monitored daily for signs of respiratory disease according to industry-standard protocols (anorexia, nasal discharge, change in respiratory pattern, rectal temperature ≥ 40°C and Whisper lung score ≥ 3) (12). Deep NP samples were collected with double-guarded sterile culture swab (Kalayjian Industries, Inc. U.S.A.) from calves diagnosed with BRD at the initial diagnosis and prior to treatment (BRD group, *n* = 9) according to published techniques (14). Equivalent NP samples were collected from clinically healthy, pen-matched controls calves (control group, *n* = 9) at the same time as the BRD-affected calves sampling. The BRD-affected calves were treated with a single dose of tilmicosin (10 mg/kg SC; Micotil, Elanco Animal Health) according to label instructions. Immediately after sample collection and treatment, each of the calves were returned to their original group pen. At day 7 post tilmicosin treatment, follow-up clinical examinations of the BRD-affected calves was performed, and disease recovery was characterized by the absence of respiratory signs, rectal temperature ≤ 39°C and Whisper lung score ≤ 2. A deep NP swab was collected from each tilmicosin-treated calf at this time (post-treatment group, *n* = 9). Following collection, all NP swabs (*n* = 27) were held on dry ice and transported to the laboratory where they were stored at −20°C pending further processing.

### DNA Extraction, 16S rRNA Gene Sequencing and Bioinformatics

Extraction of DNA was performed from all NP swabs using power® Fecal DNA isolation Kit (MO BIO Laboratories, Inc., Carlsbad, CA) following the manufacturer's instructions (15). Total DNA concentration and purity were evaluated by optical density using a NanoDrop ND-1000 spectrophotometer (NanoDrop Technologies, Rockland, DE, USA) at the wavelengths of 230, 260, and 280 nm, and the OD260/280 ratio of DNA ranged between 1.75 and 1.90. Genomic DNA was then transferred to the DNA Services lab at the W. M. Keck Center for Comparative and Functional Genomics at the University of Illinois at Urbana-Champaign for amplification and sequencing. The V1-V3 hypervariable regions of 16S ribosomal ribonucleic acid (rRNA) were amplified by Fluidigm access array amplification protocol (Fluidigm Corporation, South San Francisco, CA, USA) using the primer set F28-2-for (ACACTGACGACATGGTTCTACA) and R519-2-rev (TACGGTAGCAGAGACTTGGTCT) tagged with unique eight-base sequence barcodes. PCR reactions were performed on a Fluidigm Biomark HD™ PCR machine (Fluidigm Corporation, South San Francisco, CA, USA) using the default Access Array cycling program without imaging (Table S1). The final harvested products were quantified on a Qubit fluorometer and assessed using a Fragment Analyzer (Advanced Analytics, Ames, IA, USA) to confirm amplicon regions and sizes. The final pooled Fluidigm libraries were sequenced on the Illumina Miseq V2 platform according to the manufacturer's instructions (Illumina, San Diego, CA, USA).

The 16S rRNA gene sequences data obtained from the MiSeq sequencing were processed and analyzed with the Quantitative Insights Into Microbial Ecology (QIIME) algorithms using an operational taxonomic units (OTUs) approach (16). Sequences were quality-filtered using established guidelines (17). The open-reference OTU selection protocol (97% similarity) was conducted by QIIME using UCLUST clustering (18) and assigned taxonomy against SILVA reference database. Low abundance clusters and chimeric sequences were filtered and removed using USEARCH (19). Bacterial taxa that could not be assigned to a genus level, but were present in all NP samples, were still displayed based on the lowest taxonomic level that could be assigned to them. For subsequent bacterial diversity analysis, the OTUs table was randomly subsampled and rarefied to 3,037 sequences per sample using QIIME pipeline. The alpha diversity indices were estimated using the Chao1 richness, phylogenetic diversity (PD) whole tree and Shannon diversity indices. Fastq data obtained in the current study were uploaded to the sequence read archive on the NCBI website to make the files available for public databases with a bio-project accession number PRJNA508519.

### Statistical Analysis

Statistical analysis and graphing were performed using PAST version 3.13 and JMP® Pro 13 (SAS Institute Inc. Cary, NC, USA). For comparisons between the three groups, one-way ANOVA with all pair's comparisons using Tukey-Kramer HSD test were used to analyze data with a normal distribution and nonparametric Wilcoxon comparisons for each pair was used to analyze data that did not meet the assumptions of ANOVA. Differences between groups with *P* < 0.05 were considered statistically significant. A principal coordinate analysis (PCoA) of Bray-Curtis and Jaccard dissimilarity were performed on all samples using the relative abundance of higher taxonomic level taxa, and the significant difference between groups was analyzed using non-parametric multivariate analysis of variance (PERMANOVA) with 9999 permutations and Bonferroni corrected *P*-values in PAST version 3.13. To further quantify the overall microbial composition similarities between the different groups, unweighted pair group method with arithmetic mean (UPGMA) based on Bray–Curtis distance metrics were performed in PAST version 3.13. Finally, the Venn diagram representing the number of core shared microbiota between groups was generated.

## Results and Discussion

### Overall Taxonomic Classification and Diversity of NP Microbiota

The composition and function of the respiratory microbial ecosystem is an extensive field of research (11, 20). The nasopharyngeal microbiota is believed to be a key player in the health of the upper respiratory tract, and has been shown to be significantly modified during episodes of immunological stress and clinical respiratory disease (21). As a result of these observations, it has been suggested that disturbances in NP microbial communities may contribute to the pathophysiology of BRD in feedlot cattle (12). Although several studies have investigated the bovine NP microbiota in the predisease and disease states (22–24), little information is available on the change of NP microbiota associated with clinical recovery from BRD. To help explore this gap in knowledge, we evaluated the effect of tilmicosin treatment on the NP microbiota of BRD-affected calves during first week of clinical recovery. Sequence analysis from all NP swabs resulted in a total of 410,615 filtered sequence reads. The mean sequence reads per sample was 15,207.963 (SD, 12,104.039) and comprised a total of 604 OTUs across all samples. In terms of relative abundance, taxonomic classification of OTUs revealed a total of 14 different bacteria phyla, and 182 bacterial genera, among all samples. Similar to previous 16S rRNA gene-based studies of the NP microbiota of feedlot cattle, the most abundant bacterial phyla across all sample were *Firmicutes* (27.07%)*, Actinobacteria* (24.51%)*, Tenericutes* (16.05%), and *Proteobacteria* (14.43%) (Figure S1A) (7, 22–25). These findings are similar to those reported in studies of the nasal microbiota of pigs (26, 27) and the upper respiratory tract of humans (28). All other classified OTUs belonged to bacterial phyla and comprised <1% of the total abundance represented as others/unassigned (Figure S1A). At lower taxonomic levels, the most prevalent bacterial taxa were *Mycoplasma* (18.73%)*, Microbacteriaceae* (9.36%)*, Acinetobacter* (7.35%), and *Corynebacterium* (6.36%) (Figure S1B). Our data analysis showed a high inter-individual variability in the composition of the NP microbiota across all the individuals. This was expected, especially in the type of feedlot husbandry system used in our study, since the upper respiratory tract is constantly exposed to many and various bacteria from the surrounding environment (22). This is also compatible with other studies that have explored the multifactorial determinants (genetic, epigenetic, environmental, age, sex, and dietary) that underlie the establishment of the mucosal microbiota (29). To measure the alpha diversity of the NP microbial communities among the three groups (control, BRD, and post-treatment), we used several metrics; Shannon, Chao1, and the PD whole tree indices, as depicted in (Table 1). None of the alpha diversity indices differed significantly between groups (*p* > 0.05), although the NP microbiota from the control calves were more diverse than in the NP samples from tilmicosin-treated and BRD-affected calves. Similarly, the NP samples from tulathromycin-treated calves showed a reduction in the bacterial diversity by one-week post treatment (24). While our study did not permit longer-term evaluation of microbial biodiversity, similar studies in other species studies have shown that antimicrobial treatment is often followed by a contraction in biodiversity of some taxa that can persist for several months (30). However, in order to better understand the potential health impact of these post-treatment fluxes in community structure, it is important to explore the dynamics of these changes over time in relation to disease recovery and in association with different antimicrobial regimes. In addition, by understanding how mucosal microbiota respond to different management conditions, it should be possible to identify the mechanisms by which these communities contribute to mucosal recovery and the return of the respiratory system to a healthy state.

**Table 1:** Bacterial diversity indices (Chao1, PD whole tree and observed species) measures for the nasopharyngeal microbiota of calves.

**Bacterial diversity indices**	**Control**	**BRD**	**After treatment**	***P*-value**
Chao1 index	77.82 ± 16.40	44.34 ± 7.55	53.02 ± 13.75	0.174
PD whole tree	7.54 ± 1.10	5.76 ± 0.63	6.41 ± 0.84	0.213
Shannon index	3.66 ± 0.48	3.07 ± 0.28	3.11 ± 0.33	0.289

*The data are presented as the mean ± standard deviation. There were no statistically significant differences in different bacterial diversity indices between the different groups (p > 0.05)*.

### Comparison of NP Microbiota Across the Different Groups

**Figure 1:**
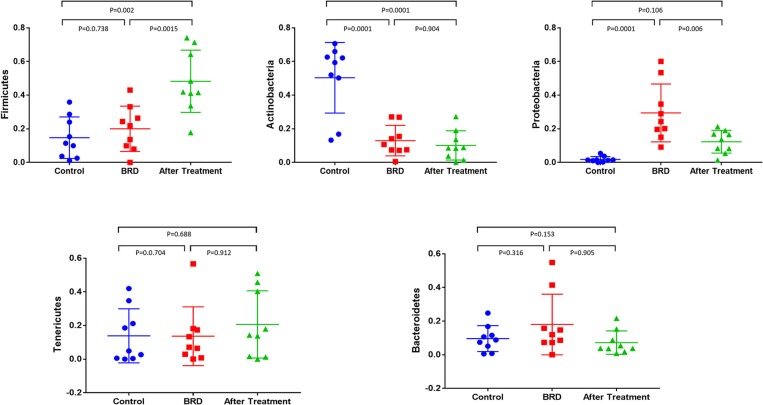
Relative abundance of bacterial 16S rRNA gene sequences at the phylum level that showed the difference between tilmicosin-treated, BRD-affected and healthy control calves. Only those bacterial phyla represent those populations that averaged more than 1% of the relative abundance across all samples when sequencing V1-V3 hypervariable regions are displayed.

**Figure 2:**
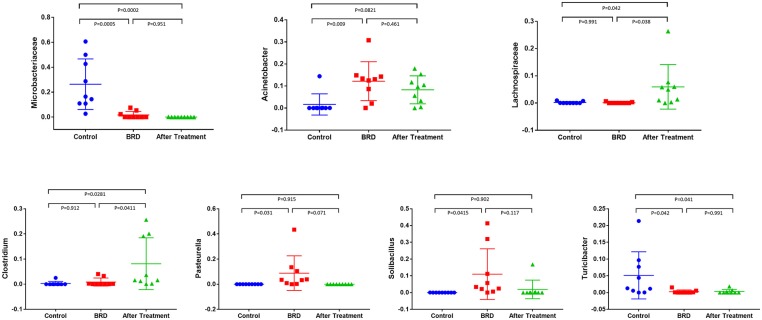
Relative abundance of bacterial 16S rRNA gene sequences at the higher taxonomic level observed in the NP swab showed the difference between tilmicosin-treated, BRD-affected and healthy control calves. Only those bacterial taxa represent those populations that averaged more than 1% of the relative abundance across all samples when sequencing V1-V3 hypervariable regions are displayed.

The dynamics of change in the NP microbiota between clinically healthy calves and those that develop BRD were reported in detail in our previous published study (12). In this study, we compared the relative abundance of the most abundant bacterial phyla that accounted for more than 1% of the total (*Firmicutes, Actinobacteria, Tenericutes, Proteobacteria*, and *Bacteroidetes*) across all three groups. In tilmicosin-treated calves, we observed a significant increase (*P* < 0.01) in the relative abundance of *Firmicutes* compared to control and BRD groups (Figure 1). Both BRD-affected and treated calves showed significant decrease in the relative abundance of *Actinobacteria* (*P* < 0.01) compared to the healthy control calves (Figure 1). The relative abundance of *Tenericutes*, and *Bacteroidetes* did not show significant changes among the groups (*P* > 0.05). At the genus level, the relative abundance of *Microbacteriaceae* (*P* < 0.001), *Acinetobacter* (*P* = 0.013), *Pasteurella* (*P* = 0.041), *Lachnospiraceae* (*P* = 0.021), *Clostridium* (*P* = 0.018), *Solibacillus* (*P* = 0.043), and *Turicibacter* (*P* = 0.026) was significantly different among the three groups (Figure 2). It is notable that the administration of a single dose of tilmicosin for BRD treatment affected the bacterial composition of the NP microbiota. Most notably, the relative abundance of *Clostridium* and *Lachnospiraceae* were significantly increased in tilmicosin-treated calves compared to control and BRD-affected calves (*P* < 0.05; Figure 2). Interestingly, it has been recently shown that an abundance of *Clostridium* species is associated with antibiotic-associated colitis and influenza in humans (31, 32), and hemorrhagic diarrhea in feedlot cattle (15). The relative abundance of *Microbacteriaceae* and *Turicibacter* was significantly decreased in tilmicosin-treated and BRD-affected calves when compared to control calves (*P* < 0.05; Figure 2). In terms of the bacterial taxa commonly associated with BRD, the overall relative abundance of *Moraxella* and *Mannheimia* was not significantly different after tilmicosin treatment. Antimicrobial administration was recently demonstrated to be efficacious in treating dairy cattle that had been experimentally challenged with *Mycoplasma* via the respiratory tract (33). Interestingly, while the changes were not statistically significant, there were general trends in the relative abundance of *Mycoplasma* and *Acinetobacter* species in the BRD-affected calves one week following treatment. A possible explanation for this finding is that because *Mycoplasma* and *Acinetobacter* population are well-known to have resistance to multiple antimicrobials (34, 35), tilmicosin treatments could have decreased the presence of other bacterial inhabitants of the nasal cavity that potentially could promote the growth of those population. This microbial reshaping due to differential sensitivity to antibiotics might explain why resilience is not complete long after antimicrobial treatment.

### Effect of Tilmicosin Treatment on the Overall NP Microbiota Composition and Core Microbiota

**Figure 3:**
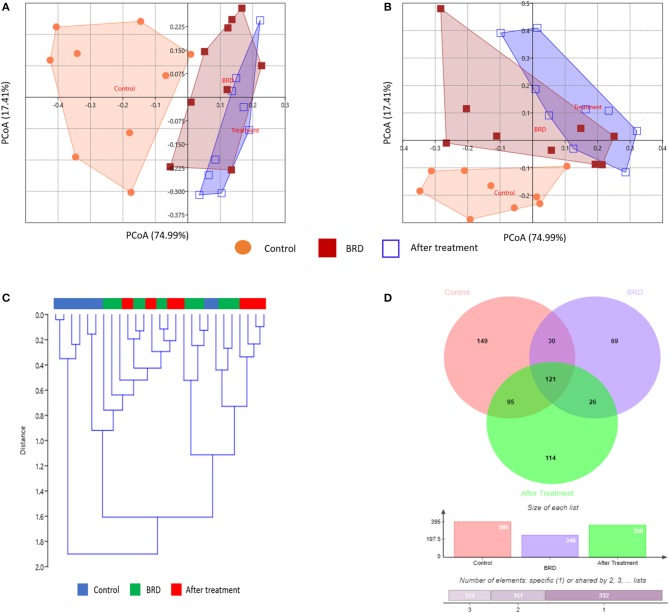
Effect of tilmicosin treatment on the overall NP microbiota composition and core microbiota. Principal coordinate analysis (PCoA) depicting the Bray-Curtis **(A)** and Jaccard dissimilarity **(B)** for the NP swab in tilmicosin-treated, BRD-affected and healthy control calves. The percent variation explained by each principal coordinate is indicated on the axes. **(C)** UPGMA clustering of bacterial taxa profiles based on the Bray-Curtis dissimilarity. The color bars above the dendrogram indicate groups of samples, control (blue), BRD (green), and after tilmicosin treatment (red). **(D)** Venn diagram depicting the common and unique OTUs among the three groups (tilmicosin-treated, BRD-affected and healthy control calves).

To evaluate the potential effect of tilmicosin antibiotic treatments on the overall NP microbiota composition of BRD-affected calves during the first week of clinical recovery, we compared the microbial community structure (beta diversity) between the three groups (Control, BRD and post-treatment) using Bray-Curtis and Jaccard dissimilarity. The PCoA plot of the Bray-Curtis and Jaccard dissimilarity revealed that the overall composition of NP microbial communities in tilmicosin-treated calves resembled that of the BRD-affected calves (PERMANOVA, *P* > 0.05; Figures 3A,B), and that both group differed significantly from pen- matched healthy calves (PERMANOVA, *P* < 0.05; Figures 3A,B). As the treatment groups did not differ significantly from the BRD groups (*P* > 0.05), it is conceivable that the tilmicosin treatment impaired recovery of the NP microbiota to a balanced homeostatic state. Unfortunately, since there was not a non-treated control group for the BRD-affected calves, the reality of a post-treatment inhibitory activity of the antimicrobial on microbiota recovery cannot be confirmed. A similar link between antimicrobial use and an altered microbial community structure in the upper respiratory tract of children up to six months after administration has been reported (36). To further evaluate the overall NP microbial similarities between the different groups, UPGMA cluster, based on Bray–Curtis distance metrics, were performed. Hierarchical clustering of the relative abundance of bacterial taxa of the NP microbiota was not evident in either the BRD or post-treatment groups. However, pen-matched clinically healthy control calves were generally clustered both closer together and further away from the BRD and post-treatment groups (Figure 3C). Additionally, a Venn diagram was generated to describe the unique and shared OTUs between the three groups (Figure 3D). With counts, the OTU distribution showed that there were 149, 69, and 114 unique OTUs identified in healthy control, BRD and post-treatment groups, respectively. Furthermore, a total of 121 OTUs, representing the core microbiota, were shared between the three groups (Figure S2). In combination, these results indicate there was little change in NP microbial communities following tilmicosin treatment, and that the initial difference in NP communities between the BRD-affected and healthy control calves remained in the early phase of clinical recovery. The absence of a non-treatment control for the BRD-affected calves prevented us from drawing any conclusions regarding the role of tilmicosin treatment in inhibiting the resolution of any BRD-related disturbances in NP microbial community structure. While tilmicosin has been implicated as being inferior to other antimicrobial treatment for control of BRD of high-risk cattle before the onset of signs of BRD (37), additional broader studies involving other types of antimicrobials, with the inclusion of the appropriate post-treatment control groups, would be required to evaluate the role of antimicrobial therapy on the rejuvenation of nasopharyngeal microbial community structure in cases of BRD.

## Conclusion

In conclusion, the overall aim of this study was to examine changes in the NP microbiome during the clinical recovery of BRD-affected calves treated with tilmicosin. It appeared that there were no significant changes in NP microbial communities following tilmicosin treatment, and that the initial differences in NP microbial communities between healthy and BRD-affected calves, remained for the duration of the early phase of clinical recovery. Given the limitations of the present study (small number of treated calves, only one type of antibiotics, short term follow-up, lack of non-treated control group) further studies are necessary to evaluate the long-term effects of antimicrobial administration upon respiratory microbiota. Understanding the potential impact of the prolonged recovery in the mucosal microbiota will be important in optimizing the use of antimicrobials in health management programs in the feedlot industry.

## Data Availability Statement

Fastq data obtained in the current study were uploaded to the sequence read archive on the NCBI website to make the files available for public databases, with a bio-project accession number PRJNA508519.

## Ethics Statement

The animal study was reviewed and approved by University of Illinois Institutional Animal Care and Use Committee (IACUC Protocol: #15064).

## Author Contributions

JL and BA designed the experiment. MZ and JL conducted the experiment. MZ performed the laboratory and data analyses. MZ and BA wrote the manuscript. All authors edited and approved the manuscript submission.

### Conflict of Interest

The authors declare that the research was conducted in the absence of any commercial or financial relationships that could be construed as a potential conflict of interest.

## Supplementary Material

Figure S1 Relative abundance of bacterial 16S rRNA gene sequences at the phylum level **(A)** and higher taxonomic level **(B)** observed in the NP swab in tilmicosin-treated, BRD-affected and healthy control calves. Only those bacterial phyla represent those populations that averaged more than 1% of the relative abundance across all samples when sequencing V1-V3 hypervariable regions are displayed.

Figure S2 Krona chart showing the relative abundance of bacterial taxa that represent the core microbiota and shared between the three groups (tilmicosin-treated, BRD-affected and healthy control calves).

Table S1 Access Array cycling program without imaging (Fluidigm Biomark HD PCR machine) for amplifying the primer/sample combinations.
