# Supplementary figures and images for: Effects of Tilmicosin Treatment on the Nasopharyngeal Microbiota of Feedlot Cattle With Respiratory Disease During the First Week of Clinical Recovery
Source: Front Vet Sci. 2020 Feb 28;7:115. doi: 10.3389/fvets.2020.00115 (PMC7059195; doi:10.3389/fvets.2020.00115)

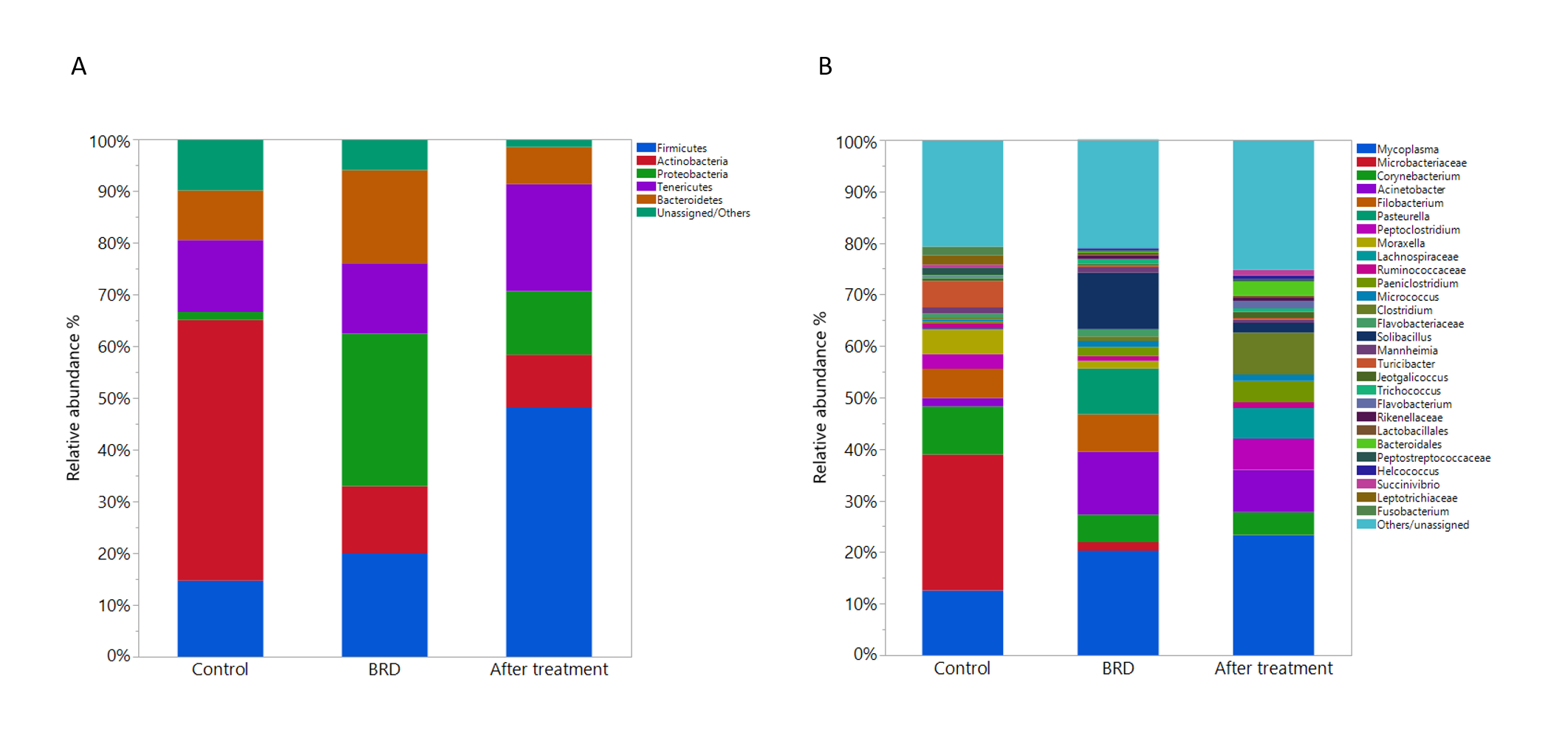

Supplement: Figure S1 — Relative abundance of bacterial 16S rRNA gene sequences at the phylum level (A) and higher taxonomic level (B) observed in the NP swab in tilmicosin-treated, BRD-affected and healthy control calves. Only those bacterial phyla represent those populations that averaged more than 1% of the relative abundance across all samples when sequencing V1-V3 hypervariable regions are displayed. [file Image_1.TIF]

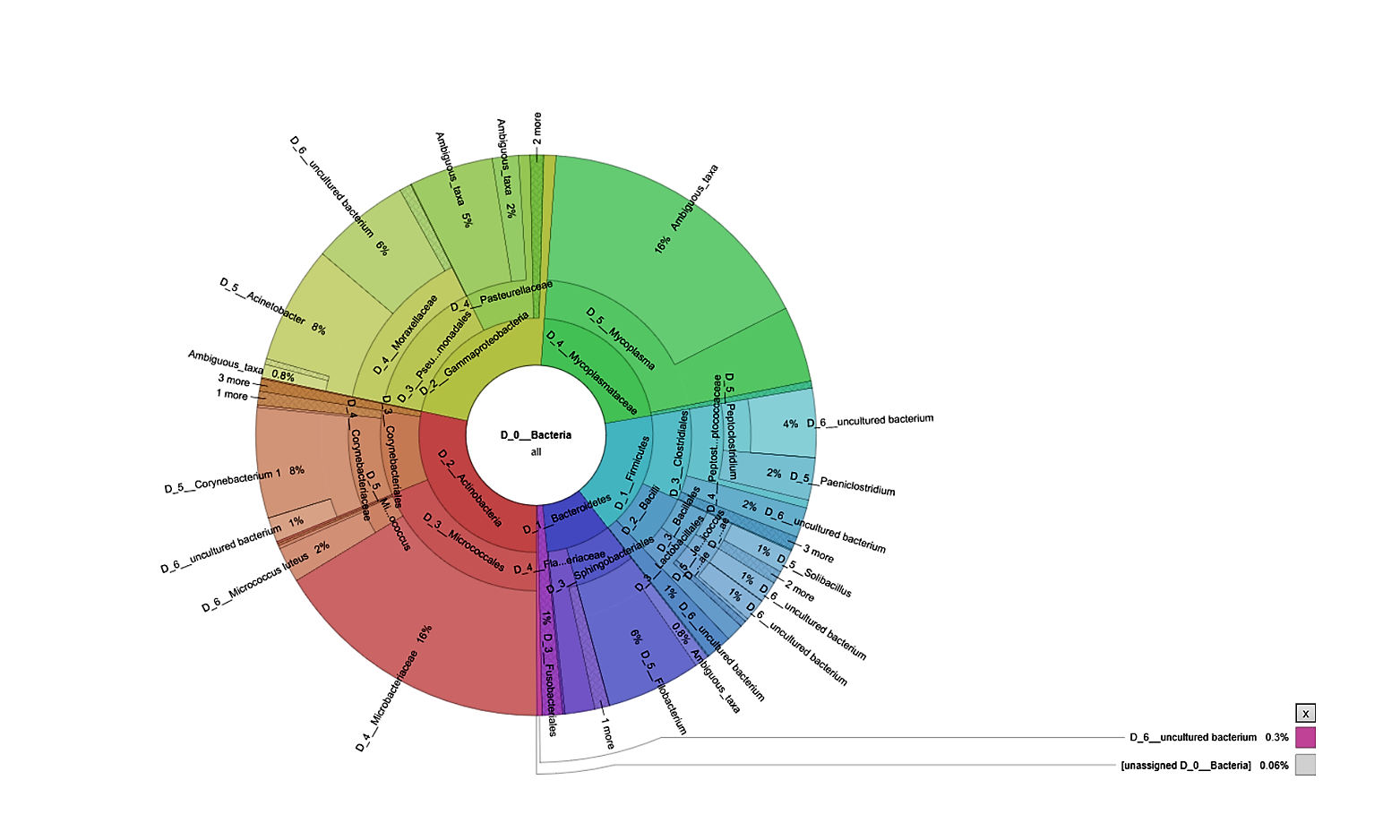

Supplement: Figure S2 — Krona chart showing the relative abundance of bacterial taxa that represent the core microbiota and shared between the three groups (tilmicosin-treated, BRD-affected and healthy control calves). [file Image_2.TIF]
