# Supplementary material for: Effects of Tilmicosin Treatment on the Nasopharyngeal Microbiota of Feedlot Cattle With Respiratory Disease During the First Week of Clinical Recovery
Source: Front Vet Sci. 2020 Feb 28;7:115. doi: 10.3389/fvets.2020.00115 (PMC7059195; doi:10.3389/fvets.2020.00115)
Supplement: Table S1 — Access Array cycling program without imaging (Fluidigm Biomark HD PCR machine) for amplifying the primer/sample combinations. [file Table_1.DOCX]

**Table S1**. Access Array cycling program without imaging (Fluidigm Biomark HD PCR machine) for amplifying the primer/sample combinations.

| **PCR Stages** | **Number of Cycles** |
| --- | --- |
| 50ºC 2 minutes | 1 |
| 70ºC 20 minutes | 1 |
| 95ºC 10 minutes   - 95ºC 15 seconds - 55ºC 30 seconds | 1 |
| 72ºC 1 minute   - 95ºC 15 seconds - 80ºC 30 seconds - 60ºC 30 seconds | 10 |
| 72ºC 1 minute   - 95ºC 15 seconds - 55ºC 30 seconds | 2 |
| 72º 1 minute   - 95ºC 15 seconds - 80ºC 30 seconds - 60ºC 30 seconds | 8 |
| 72ºC 1 minute   - 95ºC 15 seconds - 55ºC 30 seconds | 2 |
| 72ºC 1 minute   - 95ºC 15 seconds - 80ºC 30 seconds - 60ºC 30 seconds | 8 |
| 72ºC 1 minute | 5 |
